# Delta variant (B.1.617.2) sublineages do not show increased neutralization resistance

**DOI:** 10.1038/s41423-021-00772-y

**Published:** 2021-10-11

**Authors:** Prerna Arora, Amy Kempf, Inga Nehlmeier, Luise Graichen, Anzhalika Sidarovich, Martin S. Winkler, Sebastian Schulz, Hans-Martin Jäck, Metodi V. Stankov, Georg M. N. Behrens, Stefan Pöhlmann, Markus Hoffmann

**Affiliations:** 1 Infection Biology Unit, German Primate Center, Göttingen, Germany; 2 Faculty of Biology and Psychology, Georg-August-University Göttingen, Göttingen, Germany; 3 Department of Anesthesiology, University of Göttingen Medical Center, Georg-August University Göttingen, Göttingen, Germany; 4 Division of Molecular Immunology, Department of Internal Medicine 3, Friedrich-Alexander University of Erlangen-Nürnberg, Erlangen, Germany; 5 Department for Rheumatology and Immunology, Hannover Medical School, Hannover, Germany

**Keywords:** Infection, Antibodies

The emergence of SARS-CoV-2 variants threatens efforts to control the COVID-19 pandemic. At present, the global spread of the Delta (B.1.617.2) variant is responsible for a rapid increase in COVID-19 cases and hospitalizations in many countries. The variant evades neutralizing antibodies and is believed to be more transmissible and pathogenic [1–4]. Neutralizing antibodies are produced upon vaccination or infection; for the latter, it is known that levels correlate with the duration and severity of clinical symptoms [5]. Antibody evasion is caused by mutations in the viral spike protein, several of which are located in the receptor-binding domain, which is the key target of the neutralizing antibody response.

Recently, sublineages of the Delta (B.1.617.2) variant have emerged, termed Delta Plus [6], which are purported to be more transmissible. These viruses harbor the K417N mutation (Fig. 1A, B), which is also found in the Beta (B.1.351) variant and is associated with neutralization resistance [7]. Furthermore, another sublineage of the Delta variant was observed in Vietnam and might contribute to a recent surge in cases. This variant (provisionally termed Delta-V) is reported to contain mutations found in the S protein of the Alpha (B.1.1.7) variant, and it was initially proposed to be a hybrid virus. Indeed, Delta variant sequences from Vietnam and many other countries with deletions at positions H67, V70, and/or Y144 (Fig. 1A, B), also found in spike of the Alpha (B.1.1.7) variant, have been deposited in the GISAID (Global Initiative on Sharing All Influenza Data) database. Thus, sublineages of the Delta variant have arisen that might have altered biological properties and may present an increased threat to human health.

**Fig. 1:**
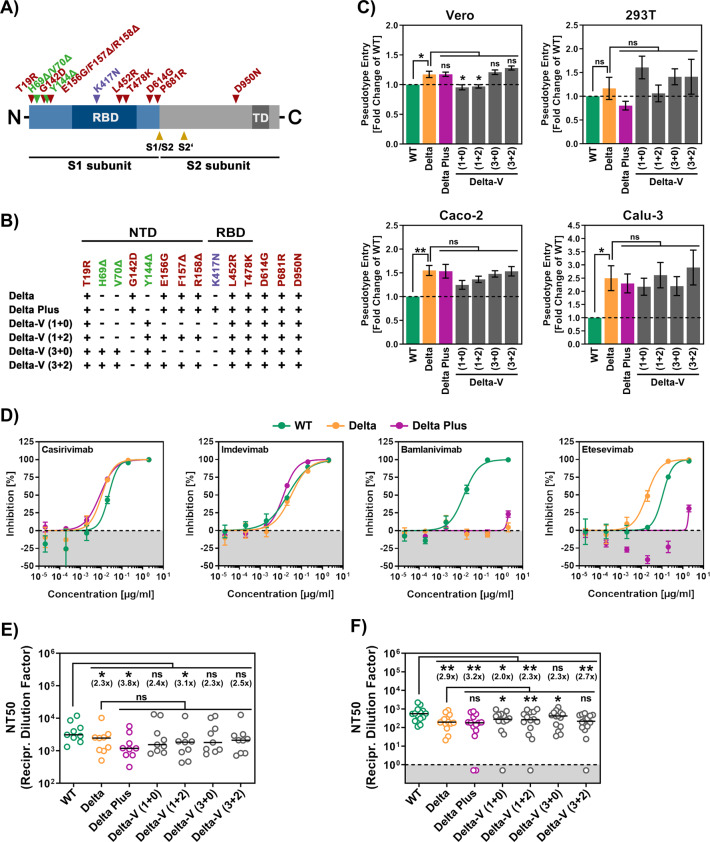
Sublineages of the Delta variant do not show increased host cell entry or resistance to neutralization by antibodies from infected or vaccinated individuals. **A** Schematic illustration of the SARS-CoV-2 S protein. Mutations associated with the Delta variant (B.1.617.2) are highlighted in red. Mutation K417N, specific for variant Delta Plus, is marked in purple; mutations identical to those found in the Alpha variant (B.1.1.7) are labeled in green (RBD, receptor-binding domain; TD, transmembrane domain). **B** A summary of S protein variants under study and their respective mutations is given below the S protein scheme (NTD, N-terminal domain). **C** Particles pseudotyped with the indicated S proteins were inoculated onto four different cell lines, and transduction efficiency was quantified by measuring virus-encoded luciferase activity in cell lysates at 16–18 h post transduction. Presented are average (mean) data from six biological replicates (each conducted with technical quadruplicates) for which transduction was normalized against wild-type (WT) SARS-CoV-2 S (=1). Error bars indicate the standard error of the mean (SEM). **D** Particles pseudotyped with WT (green), Delta (B.1.617.2) variant (yellow) or Delta Plus variant (purple) S proteins were incubated for 30 min at 37 °C in the presence of the indicated monoclonal antibodies before being inoculated onto Vero cells. Transduction efficiency was quantified as described above. For normalization, WT S protein-driven entry in the absence of monoclonal antibody was set as 0% inhibition. The average of four technical replicates is shown. Error bars indicate the standard deviation (SD). **E**, **F** Particles bearing the indicated S proteins were incubated for 30 min at 37 °C in the presence of different dilutions of convalescent plasma (**E**) or vaccinee serum (**F**) before being inoculated onto Vero cells. Transduction efficiency was quantified as stated above and used to calculate the plasma/serum dilution factor that leads to a 50% reduction in transduction (neutralizing titer 50, NT50). Data for nine convalescent plasma samples and fourteen vaccination serum samples are presented. Black lines indicate the median, and numbers in brackets represent the fold change in NT50 compared to WT SARS-CoV-2 S (dashed line = detection limit). Statistical significance was analyzed by two-tailed Student’s *t* test (^ns^*p* > 0.05; *p ≤ 0.05; **p ≤ 0.01; ***p ≤ 0.001)

We sought to determine whether Delta Plus and Delta-V differ from the Delta variant regarding cell entry and neutralization sensitivity by using rhabdoviral pseudotypes, which are adequate models for cell entry of SARS-CoV-2, and previously described monoclonal antibodies and sera/plasma from infected or BNT162b2/Comirnaty vaccinated individuals [8, 9]. The Delta Plus and Delta-V variants entered the Vero and 293T kidney-derived cell lines with similar efficiency as the Delta variant and WT virus (Wuhan/Hu-1/2019 isolate with the D614G mutation) (Fig. 1C). Furthermore, entry of the Delta variant into the lung- and colon-derived cell lines Calu-3 and Caco-2 was enhanced, in keeping with a previous study [8]; an increase in entry efficiency was also observed for the Delta Plus and Delta-V variants (Fig. 1C). The Delta variant was resistant to neutralization by the monoclonal antibody bamlanivimab, whereas Delta Plus was resistant to both bamlanivimab and etesevimab (Fig. 1D), with resistance to the latter most likely due to the K417N mutation [10]. Finally, the Delta variant showed reduced neutralization by antibodies induced by SARS-CoV-2 infection and BNT162b2/Comirnaty vaccination, in agreement with previous findings and in line with data for its parental lineage B.1.617 [2–4, 8, 9, 11] and neutralization of the Delta Plus and Delta-V variants was reduced to similar levels (Fig. 1E, F).

Our results reveal no appreciable differences between host cell entry and neutralization sensitivity of the Delta variant and its sublineages, with the notable exception of the Delta Plus variant being resistant to both bamlanivimab and etesevimab, which are used in cocktails for COVID-19 treatment. Although T-cell responses were not analyzed in the present study and confirmation of the data using authentic virus and primary cells is pending, our results suggest that neither the Delta Plus nor the Delta-V variant is associated with an increased threat to convalescent or BNT162b2/Comirnaty vaccinated patients compared to the founder virus. Finally, it is noteworthy that heterologous ChAdOx1 nCov-19/BNT162b2 vaccination might provide particularly robust protection against these viruses [12].

## Supplementary information

Supplemental Material
