# Supplementary material for: Delta variant (B.1.617.2) sublineages do not show increased neutralization resistance
Source: Cell Mol Immunol. 2021 Oct 11;18(11):2557–9. doi: 10.1038/s41423-021-00772-y (PMC8503871; doi:10.1038/s41423-021-00772-y)
Supplement: Supplementary file 1 — Supplemental Material [file 41423_2021_772_MOESM1_ESM.docx]

**Supplementary Material**

**Delta variant (B.1.617.2) sub-lineages do not show increased neutralization resistance**

Prerna Arora^1,2^, Amy Kempf^1,2^, Inga Nehlmeier^1^, Luise Graichen^1,2^, Anzhalika Sidarovich^1,2^, Martin S. Winkler^3^, Sebastian Schulz^4^, Hans-Martin Jäck^4^, Metodi V. Stankov^5^, Georg M. N. Behrens^5^, Stefan Pöhlmann^1,2,^, Markus Hoffmann^1,2^

^1^Infection Biology Unit, German Primate Center, Kellnerweg 4, 37077 Göttingen, Germany

^2^Faculty of Biology and Psychology, Georg-August-University Göttingen, Wilhelmsplatz 1, 37073 Göttingen, Germany

^3^Department of Anesthesiology, University of Göttingen Medical Center, Göttingen, Georg-August University of Göttingen, Robert-Koch-Straße 40, 37075 Göttingen, Germany

^4^Division of Molecular Immunology, Department of Internal Medicine 3, Friedrich-Alexander University of Erlangen-Nurnberg, Glückstraße 6, 91054 Erlangen, Germany

^5^Department for Rheumatology and Immunology, Hannover Medical School, Carl-Neuberg-Straße 1, 30625 Hannover, Germany

E-mail: Markus Hoffmann (mhoffmann@dpz.eu), Stefan Pöhlmann (spoehlmann@dpz.eu)

# Material and Methods

**Cell culture**

293T (human, female, kidney; ACC-635, DSMZ; RRID: CVCL_0063) and Vero cells (African green monkey kidney, female, kidney; CRL-1586, ATCC; RRID: CVCL_0574, kindly provided by Andrea Maisner) were cultured in Dulbecco’s modified Eagle medium (DMEM, PAN-Biotech) supplemented with 10% fetal bovine serum (FBS, Biochrom), 100 U/ml penicillin and 0.1 mg/ml streptomycin (pen/strep) (PAN-Biotech). Additionally, Calu-3 (human, male, lung; HTB-55, ATCC; RRID: CVCL_0609, kindly provided by Stephan Ludwig) and Caco-2 cells (human, male, colon; HTB-37, ATCC, RRID: CVCL_0025) were cultured in minimum essential medium (MEM, GIBCO) supplemented with 10% FBS, 1% pen/strep, 1x non-essential amino acid solution (from 100x stock, PAA) and 1 mM sodium pyruvate (PAN-Biotech). All cell lines were incubated at 37 °C in a humidified atmosphere containing 5% CO_2_. Cell lines were validated by STR-typing, amplification and sequencing of a fragment of the cytochrome c oxidase gene, microscopic examination and/or according to their growth characteristics. Furthermore, all cell lines were regularly tested for mycoplasma contamination.

**Expression plasmids**

Plasmids pCAGGS-DsRed (1), pCAGGS-VSV-G (vesicular stomatitis virus glycoprotein) (2), pCG1-WT SARS-CoV-2 S (codon-optimized, based on the Wuhan/Hu-1/2019 isolate, contains D614G exchange; with a C-terminal truncation of 18 amino acid, GISAID Accession ID: EPI_ISL_425259) (3) and Delta (B.1.617.2, GISAID Accession ID: EPI_ISL_1921353) S (4) have been previously described. To generate the expression plasmids for the S proteins of SARS-CoV-2 variants Delta Plus and Delta-V the mutations were introduced into the Delta S sequence by overlap extension PCR and the respective PCR fragments inserted into plasmid pCG1 (kindly provided by Roberto Cattaneo, Mayo Clinic College of Medicine, Rochester, MN, USA), using restriction sites BamHI and XbaI. The integrity of all PCR-amplified sequences was verified by sequence analyses performed by a commercial service provider (Microsynth SeqLab).

**Production of rhabdoviral pseudotypes and transduction of target cells**

Viral particles pseudotyped with the SARS-CoV-2 S protein were produced as described (5, 6). In brief, 293T cells were transfected with plasmids encoding S protein, VSV-G or empty plasmid (control) using the calcium phosphate method. At approximately 30 h posttransfection, cells were inoculated with VSV-G-transcomplemented VSV*ΔG(FLuc), a replication-deficient vesicular stomatitis virus (VSV) that lacks the genetic information for its own glycoprotein (VSV-G) and instead codes for two reporter proteins, enhanced green fluorescent protein (eGFP) and firefly luciferase (kindly provided by Gert Zimmer) (6). After 1 h of incubation, the inoculum was removed and cells were washed with phosphate-buffered saline (PBS). Thereafter, to neutralize residual input virus, all cells received DMEM medium containing anti-VSV-G antibody (culture supernatant from I1-hybridoma cells; ATCC no. CRL-2700) except for cells expressing VSV-G, which received medium without antibody. After an incubation period of 16-18 h, the culture supernatant was harvested, clarified from cellular debris by centrifugation at 4,000 x g for 10 min, aliquoted and stored at -80 °C or directly used for transduction experiments. For transduction, target cells seeded in 96-well plates were inoculated with equal volumes of pseudotypes and transduction efficiency was evaluated at 16-18 h post transduction by measuring luciferase activity in cell lysates. For this, cells were lysed in PBS containing 0.5% Triton X-100 (Carl Roth) for 30 min at room temperature. Subsequently, cell lysates were transferred into white 96-well plates and mixed with luciferase substrate (Beetle- Juice, PJK) before luminescence was measured using a luminometer (Hidex).

**VSV pseudotype-based neutralization assay**

Collection of serum and plasma samples and corresponding patient information have been described before (3, 4, 7). All serum and plasma samples were heat-inactivated at 56 °C for 30 min and pre-screened for their ability to neutralize transduction of Vero cells by particles pseudotyped with WT SARS-CoV-2 S. Neutralization assays were conducted as described (4, 7). In brief, S protein bearing particles were pre-incubated for 30 min at 37 °C with different concentrations of Casirivimab, Imdevimab, Bamlanivimab, Etesevimab, or unrelated control IgG (2, 0.2, 0.02, 0.002, 0.0002, 0.00002 μg/ml). Alternatively, pseudotyped particles were pre-incubated with different dilutions (1:25, 1:100, 1:400, 1:1,600 and 1:6,400) of convalescent plasma or serum from BNT162b2/Comirnaty vaccinated individuals. Following incubation, mixtures were inoculated onto Vero cells with particles incubated with medium alone serving as control. Transduction efficiency was determined at 16-18 h postinoculation as described above.

**Statistical analysis**

Data were analyzed using Microsoft Excel (as part of the Microsoft Office software package, version 2019, Microsoft Corporation) and GraphPad Prism 8 version 8.4.3 (GraphPad Software). Statistical significance was tested by two-tailed Students t-test. Only p-values of 0.05 or lower were considered statistically significant (p > 0.05, not significant [ns]; p ≤ 0.05, *; p ≤ 0.01, **; p ≤ 0.001, ***).

**REFERENCES**

1. Hoffmann M, Kleine-Weber H, Schroeder S, Kruger N, Herrler T, Erichsen S, et al. SARS-CoV-2 Cell Entry Depends on ACE2 and TMPRSS2 and Is Blocked by a Clinically Proven Protease Inhibitor. Cell. 2020;181(2):271-80 e8.

2. Brinkmann C, Hoffmann M, Lubke A, Nehlmeier I, Kramer-Kuhl A, Winkler M, et al. The glycoprotein of vesicular stomatitis virus promotes release of virus-like particles from tetherin-positive cells. PLoS One. 2017;12(12):e0189073.

3. Hoffmann M, Arora P, Gross R, Seidel A, Hornich BF, Hahn AS, et al. SARS-CoV-2 variants B.1.351 and P.1 escape from neutralizing antibodies. Cell. 2021;184(9):2384-93 e12.

4. Arora P, Kempf A, Nehlmeier I, Sidarovich A, Krüger N, Graichen L, et al. Increased lung cell entry of B.1.617.2 and evasion of antibodies induced by infection and BNT162b2 vaccination. bioRxiv. 2021:2021.06.23.449568.

5. Kleine-Weber H, Elzayat MT, Wang L, Graham BS, Muller MA, Drosten C, et al. Mutations in the Spike Protein of Middle East Respiratory Syndrome Coronavirus Transmitted in Korea Increase Resistance to Antibody-Mediated Neutralization. J Virol. 2019;93(2).

6. Berger Rentsch M, Zimmer G. A vesicular stomatitis virus replicon-based bioassay for the rapid and sensitive determination of multi-species type I interferon. PLoS One. 2011;6(10):e25858.

7. Hoffmann M, Hofmann-Winkler H, Kruger N, Kempf A, Nehlmeier I, Graichen L, et al. SARS-CoV-2 variant B.1.617 is resistant to bamlanivimab and evades antibodies induced by infection and vaccination. Cell Rep. 2021;36(3):109415.
